# Comments on Delinquent Subscribers

**Published:** 1891-08

**Authors:** John Hall

**Affiliations:** Victoria, British Columbia


### COMMENTS ON DELINQUENT SUBSCRIBERS.

EDITORS OF THE HOMŒOPATHIC PHYSICIAN:

I am very much surprised that you have found it necessary to issue a special appeal to those subscribers who take THE HOMŒOPATHIC PHYSICIAN, but who do not pay their dues. Indeed I can hardly realize that any one can send for and peruse *such* a journal, the very first of its kind issued in this or any other country for the exposition of *Pure Homœopathy* and not willingly pay for it. Started by such *men* as Lippe, Hering, Wells, etc., in the midst of continuous and severe opposition growing worse and more subtle and oily, amid rankness in therapeutics, it has, nevertheless, maintained its course some ten years. I say it is hard to realize that some are so lost to all sense of obligation that they are willing to avail themselves of this exponent while remaining unwilling to remunerate the editors and publishers with the very small fee which they so justly earn.

You are at liberty to publish this letter, though I am very far from wishing to offend any subscriber, rather thinking that the wants of the editors have been overlooked amid the pressure of daily practice; it being *impossible* to be a *genuine consistent homœopath without recognizing the claims of this Journal.* Let me hope that you will at once receive their remittances with as many more subscriptions as they can make, and go on with the publication of it. Each number contains, to every thoughtful physician, suggestions and experiences worth more than a year's subscription. To fail in supporting it, and thus allowing it to drop out of its career would prove a *lasting* disgrace to our school, causing very many *sad hearts* in our ranks, and rejoicings in the camps of the enemy the world over. That excellent institution, the Philadelphia Post-Graduate School, where pure Homœopathy is truly taught as a science, in contrast with so many of our colleges which know and teach so little of it while *calling* themselves "Homœopathic" and preaching both Eclectism and spurious Homœopathy: *That* school about which too much cannot be said in its favor, will soon cease to exist as the exponent of true Homœopathy if THE HOMŒOPATHIC PHYSICIAN be allowed to die. May God arrest this event, giving to the editors such support that they will not be driven as a necessity to advertisements of doubtful quality by which so many journals are sustained.

Very truly yours, JOHN HALL.

VICTORIA, BRITISH COLUMBIA, April 22d, 1891.

